# Chronic Inflammation and γδ T Cells

**DOI:** 10.3389/fimmu.2016.00210

**Published:** 2016-05-27

**Authors:** Nathan S. Fay, Emily C. Larson, Julie M. Jameson

**Affiliations:** ^1^Department of Biological Sciences, California State University, San Marcos, San Marcos, CA, USA

**Keywords:** γδ T cells, inflammation, epithelia, intestine, obesity, diabetes, cytokines, lungs

## Abstract

The epithelial tissues of the skin, lungs, reproductive tract, and intestines are the largest physical barriers the body has to protect against infection. Epithelial tissues are woven with a matrix of immune cells programed to mobilize the host innate and adaptive immune responses. Included among these immune cells are gamma delta T lymphocytes (γδ T cells) that are unique in their T cell receptor usage, location, and functions in the body. Stress reception by γδ T cells as a result of traumatic epithelial injury, malignancy, and/or infection induces γδ T cell activation. Once activated, γδ T cells function to repair tissue, induce inflammation, recruit leukocytes, and lyse cells. Many of these functions are mediated *via* the production of cytokines and growth factors upon γδ T cell activation. Pathogenesis of many chronic inflammatory diseases involves γδ T cells; some of which are exacerbated by their presence, while others are improved. γδ T cells require a delicate balance between their need for acute inflammatory mediators to function normally and the detrimental impact imparted by chronic inflammation. This review will focus on the recent progress made in understanding how epithelial γδ T cells influence the pathogenesis of chronic inflammatory diseases and how a balance between acute and chronic inflammation impacts γδ T cell function. Future studies will be important to understand how this balance is achieved.

## γδ T Cells Play Regulatory Roles in a Variety of Epithelial Tissues

### Skin

**Figure 1:**
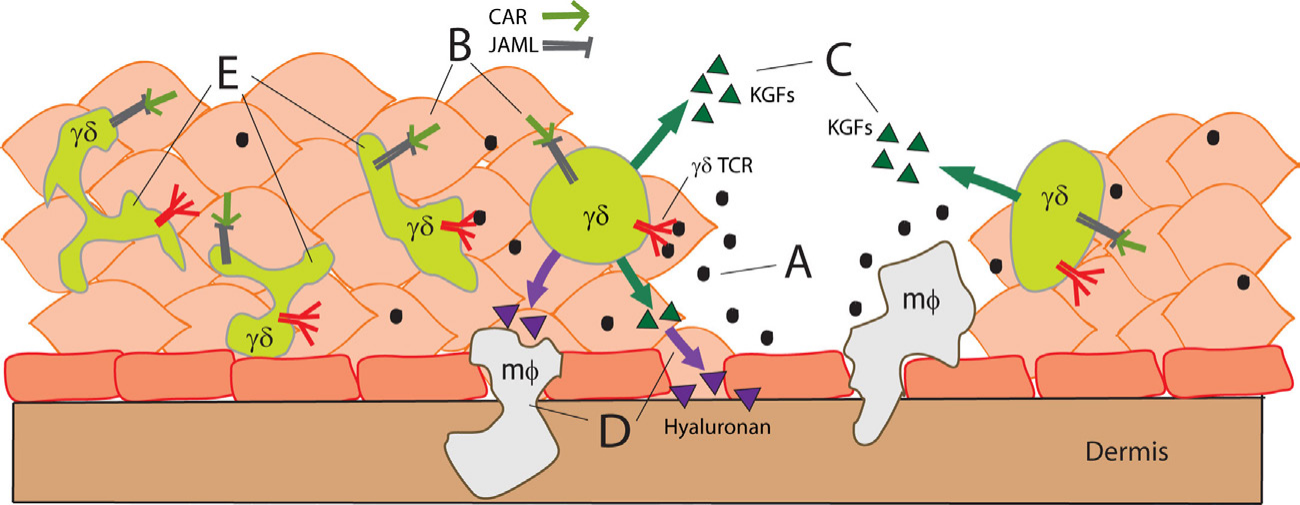
**Stress-related signals activate γδ T cells following trauma to epithelial tissues (A)**. CAR–JAML interaction between keratinocytes and γδ T cells **(B)** costimulates γδ T cell activation. Activated γδ T cells secrete growth factors to initiate tissue repair **(C)** and trigger macrophage (mφ) recruitment to assist in inflammation **(D)**. γδ T cells proximal to the wound site have fewer dendrites and appear more rounded **(E)**.

Resting epidermal gamma delta T lymphocytes (γδ T cells) exhibit a dendritic morphology and hence are referred to as dendritic epidermal T cells (DETCs). DETCs express canonical γδ T cell receptors that recognize an as yet unidentified antigen without requiring classical major histocompatibility comple (MHC) presentation (1, 2). Stress, trauma, tumorigenesis, and/or some infections (3) of local keratinocytes induce activation of the γδ T cell receptor (TCR) causing DETCs to round-up and produce growth factors (4–6). In order for DETCs to recognize local stress signals and become activated, additional costimulatory mechanisms must be triggered. These signals include (1) ligation of the cluster of differentiation 100 (CD100) receptor by Plexin B2 and (2) ligation of junction adhesive molecule-like protein (JAML) by coxsackieviruses and adenovirus receptor (CAR) (7, 8). Figure 1 illustrates this interaction by showing that stress-related signals from damage to epithelial tissues (Figure 1A) and costimulation between keratinocytes and γδ T cells (Figure 1B) trigger growth factor production (Figure 1C) and chemokine secretion (Figure 1D) that ultimately assist in wound repair. Using a γδ TCR tetramer, it has been shown that the antigen recognized by DETCs is upregulated several hours post-wounding just prior to DETC activation and rounding (6).

Dendritic epidermal T cells participate in wound healing by releasing cytokines, chemokines, and growth factors that regulate leukocyte recruitment, inflammation, and keratinocyte proliferation (4, 9). It is known that inflammation in burned epithelial tissues stimulates the activity of inducible nitric oxide synthase (iNOS) leading to increased production of nitric oxide – a potent vasodilatory cytokine (10). Cytokine production was examined when an epidermal burn was applied to mice with or without γδ T cells (11). In each of these strains, a peak in production was noted at 3 days post-burn, which coincided with peak tissue inflammation. During this peak in inflammation, wild-type mice exhibited an 18-fold increase in iNOS expression when compared to unburned mice, while iNOS expression had increased only 20% in γδ T cell-deficient mice (TCRδ^−/−^ mice) (11). Mice administered an iNOS inhibitor failed to adequately repair burned epithelial tissues, suggesting a major role for DETCs in iNOS production in wound inflammation (11).

A subsequent study found that γδ T cells influence the recruitment of Th1, Th2, and Th17 cells to a burn site (12). When mice were subjected to an epidermal burn, Rani et al. observed a significant influx in cluster of differentiation 3 (CD3+) T cells at the burn site as compared with unburned sham mice. At 7 days post-burn, γδ T cells expressing IFN-γ increased 3-fold, while IL-10- and IL-17-producing γδ T cells increased 9-fold and 20-fold, respectively. These regulatory roles of γδ T cells in the skin suggest that inflammation can be skewed to improve or impair wound healing (12). It is unknown if epidermal or dermal γδ T cells are responsible, but it may be a combination of both. γδ T cell regulatory functions are likely to be altered in environments with preexisting chronic inflammation as in obesity or aging.

### Intestine

Intraepithelial γδ T cells comprise approximately 50% of total CD3+ T cells in the small intestine. The role of γδ intraepithelial lymphocytes (IELs) in the intestine has been somewhat controversial. Some populations of γδ IELs protect intestinal epithelial cells through the production of growth factors, while other γδ IEL populations inflict potentially detrimental effects. Unlike the anchored γδ T cells of the skin, γδ IELs in the intestine have the ability to migrate throughout the intestinal epithelia and the lamina propria by an occludin-dependent mechanism – allowing γδ T cells to survey the entire villous epithelium (13, 14). Knockout of occludin in mouse γδ IELs *in vivo* leads to a deficiency in γδ T cell mobility within the intestinal epithelium, and thus a reduction in intestinal γδ IEL number (14). The presence of TNF-α also seems to limit γδ IEL migration patterns; however, it does so while increasing γδ IEL motility within the epithelium (14). This may represent an acute inflammatory response that serves to focus γδ IELs to the site of damage or infection.

Intestinal γδ IELs regulate tissue homeostasis and repair in the epithelium. When activated, γδ T cells in the intestinal epithelium produce keratinocyte growth factor 1 (KGF-1, also known as FGF-7) (14). KGF-1 acts on the intestinal epithelium to induce epithelial cell proliferation and repair of the epithelium, as needed (15, 16). Mice lacking γδ T cells, TCRδ^−/−^ mice, experience increased susceptibility to DSS-induced colitis and a reduced ability to repair damaged epithelia compared to wild-type mice (15, 16). γδ T cells also participate in the maintenance of other intestinal features that regulate barrier function. In TCRδ^−/−^ mice, mucus-secreting goblet cells are significantly reduced in number and the intestinal crypt lengths are shortened (16). Thus, γδ T cells are important for the maintenance of the intestinal barrier and restoration upon damage.

Inflammatory mediators are likely to impact γδ IEL function in the intestine. TNF-like protein 1A (TL1A) is a pro-inflammatory cytokine from the TNF cytokine family, which is found in high concentrations in mouse models of inflammatory bowel disease (IBD) (17). TL1A knockout mice exhibit fewer γδ IELs as compared to wild-type mice (17). It is suggested that TL1A regulates the infiltration and maintenance of γδ IELs in the intestinal epithelium (17). It is unknown how the chronic production of inflammatory mediators would impact γδ IEL function or maintenance in the intestine.

### Lungs

Pulmonary epithelial γδ T cells also contribute to the maintenance of barrier integrity and to mucosal immunity in the lungs. At birth, mouse Vγ6+ T cells from the thymus seed the lung; however, at 3 weeks of age, Vγ4+ T cells become more prominent, followed by Vγ5+ and Vγ7+ T cells (18). γδ T cells preferentially colonize pulmonary epithelial tissues to promote epithelial growth, regulate allergic airway hyper-reactivity by Th2 cells, regulate inflammatory responses during infection, and more (18). When traumatic hemorrhage is induced in the lungs of mice, pulmonary γδ T cells regulate the infiltration of both αβ T cells and myeloid-derived suppressor cells into the pulmonary epithelium while also increasing their own numbers (19). Recent studies have focused on the IL-17-producing ability of lung γδ T cells in regulating the outcome of disease.

IL-17-producing pulmonary intraepithelial γδ T cells require microbiota to function and confer antitumor activity (20). Cheng et al. examined γδ T cell populations in mice treated with antibiotics that were then challenged by an induction of Lewis lung carcinoma or melanoma. Treatment with antibiotics reduced resident populations of bacteria in the lungs of the mice; however, mice receiving the antibiotic treatment also experienced a decreased number of pulmonary epithelial IL-17-producing γδ T cells (20). The study found that regardless of which bacterial population was inhibited in the lungs of these mice, the absence of commensal bacteria was enough to inhibit IL-17-producing γδ T cell antitumor capabilities in the pulmonary epithelium. As a result, these antibiotic-treated mice became susceptible to lung tumors. The antitumor response could be restored when the mice that were treated with antibiotics received a transplant of pulmonary intraepithelial γδ T cells from healthy mice never treated with antibiotics (20).

The mechanism by which γδ T cells prevent human lung cancer involves the synthesis of ligands from the TNF family of cytokines. Tumor necrosis factor-related apoptosis-inducing ligand (TRAIL) synthesis by γδ T cells in the lungs trigger lysis of cancer cells expressing TRAIL-R2, or occasionally TRAIL-R1 (21). Stimulation with NK receptor group 2 member D (NKG2D) increases TRAIL production and enhances the ability of γδ T cells to kill lung cancer cells (21). TNFα induces the expression of IL-6 and IL-23, which are required for full IL-17 expression in γδ T cells of the lung. When mice deficient in TNFα are challenged with ozone, to induce pulmonary damage, they exhibit decreased IL-17 production by γδ T cells and a reduced inflammatory response (22).

Recent studies have increased our understanding of how IL-17 production in γδ T cells is regulated. Early growth response 3 (Egr3) is a transcription factor activated by mitogens that negatively regulates the activation of T cells and renders the Th1 T cell population unresponsive (23). Though Egr3 limits Th1 cell activation and responses, it promotes γδ T cell growth and pro-inflammatory IL-17 production. Mice engineered to overexpress Egr3 were found to experience a fivefold increase in splenic and pulmonary γδ T cell numbers compared to wild-type mice, indicating that Egr3 plays a crucial role in maintaining γδ T cell populations (23). Mice overexpressing Egr3 were then given pulmonary fibrosis to study inflammation and IL-17 expression. It was found that mice with Egr3 overexpression exhibit greater inflammation and fibrosis of their lungs and consequently experience a lower rate of survival than wild-type mice expressing normal levels of Egr3 (23). This suggests that IL-17-producing pulmonary γδ T cells perform a key pro-inflammatory role that can be harmful. Therefore, IL-17-producing pulmonary γδ T cells govern a fine balance between the underproduction and overproduction of inflammatory responses in the lungs.

## γδ T Cells in Chronic Inflammatory Diseases

### Obesity and Type 2 Diabetes

**Figure 2:**
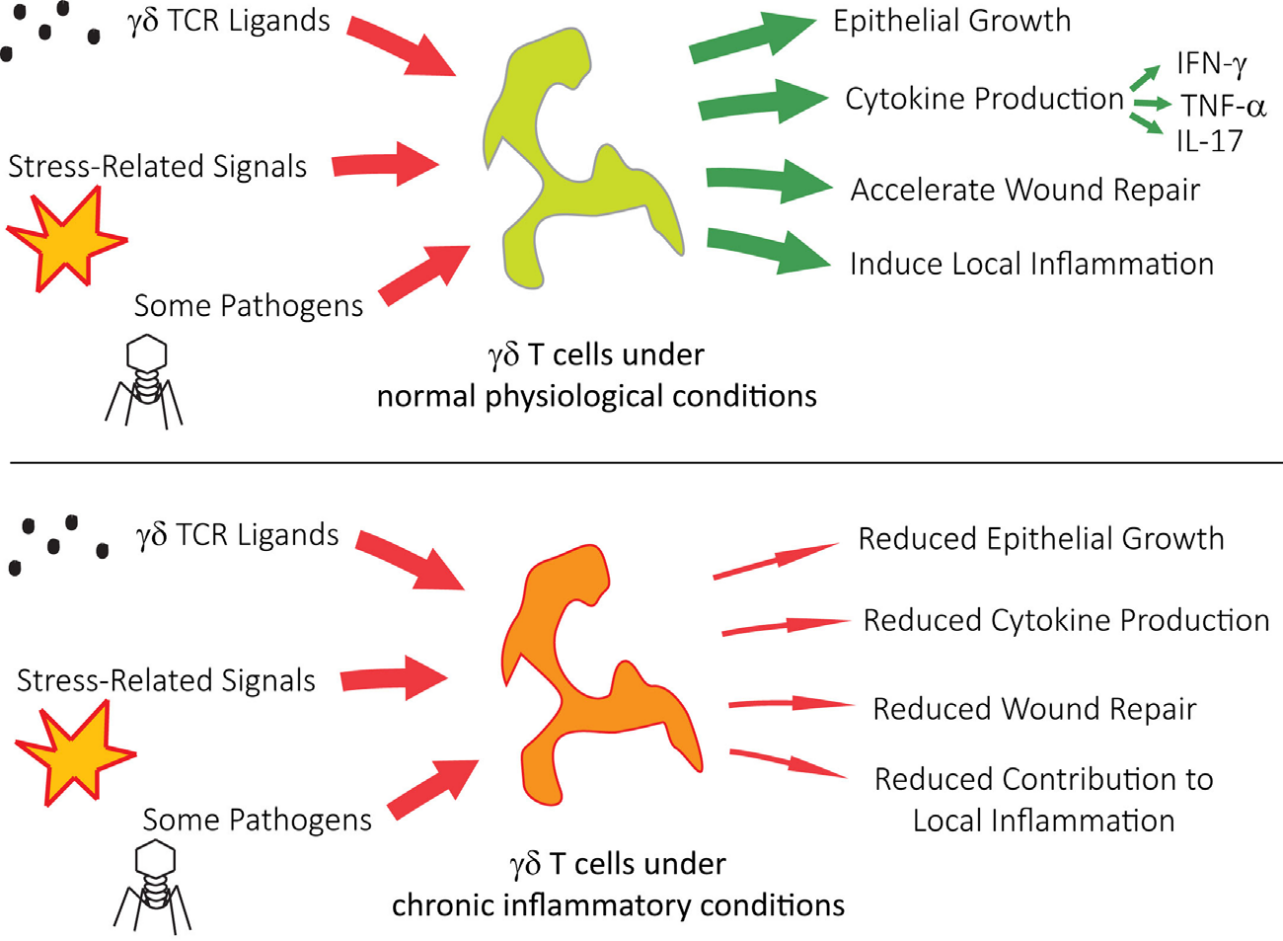
**γδ T cells under chronic inflammatory conditions show reduced activity, contributing to multiple pathophysiological issues**.

Human Vγ9Vδ2 T cells (also known as Vγ2Vδ2 T cells) are found predominantly in blood and lymphoid tissues, where some of the many varieties of Vγ9Vδ2 T cells act as first responders to pathogens (24–26), cell stress (26), and cancer (25, 27). Upon activation, Vγ9Vδ2 T cells respond by producing cytokines, performing cytolysis, and proliferating. Obese individuals are at great risk for systemic inflammation, which can lead to type 2 diabetes, cardiac disease, and increased susceptibility to infection (28). Once systemic inflammation becomes chronic, obese patients exhibit a reduced number of Vγ9Vδ2 T cells. This reduction is inversely proportionate to their body mass indices (29). The remaining Vγ9Vδ2 T cells are significantly reduced in their ability to secrete IFN-γ during viral infections like influenza and possess a differential bias toward mature T effector memory cluster of differentiation 45RA (CD45RA^+^) cells. As summarized in Figure 2, chronic inflammatory conditions pose significant pathophysiological implications on γδ T cell function. Recent *in vitro* experiments are already showing that it is possible to rescue Vγ9Vδ2 T cell function with the addition of IL-2, which restores Vγ9Vδ2 T cell cytokine production (29).

Studies in murine models of obesity have identified similarly reduced γδ T cell numbers in the skin of obese mice (30). In leptin receptor-deficient (*db/db*) mice, epidermal γδ T cell numbers were not significantly different from their *db/*+ lean counterparts until 6 weeks of age. For the next 8 weeks, the γδ T cell numbers in *db/db* mice fell to half the number of γδ T cells found in *db/*+ mice (30). A concurrent wound healing study found that the skin γδ T cells of obese *db/db* mice exhibit a reduced ability to produce cytokines and growth factors at the wound site (26). The dysfunctional γδ T cells in wounded obese *db/db* mice expressed substantially less TGF-β than lean wounded mice. Most remarkably, when systemic TNFα levels were reduced in obese *db/db* mice using neutralizing anti-TNF-α antibodies, their epidermal γδ T cells demonstrated a significant improvement in function (30). Similarly, γδ T cells in the lungs of obese mice produce less IL-17 than γδ T cells in the lungs of their lean counterparts in response to ozone (22). Regardless of whether obesity in mice is caused by leptin receptor deficiency (*db/db*) or high fat diet (HFD – 60% kcal from fat), the loss of skin γδ T cells in obesity results in a reduction in keratinocyte number in the basal layer due to premature keratinocyte differentiation and proliferation (31). Together, these studies reveal the similarly detrimental impact of chronic inflammation in obesity on γδ T cell populations in humans and in mouse models of obesity.

### Psoriasis

Psoriasis is a hyperproliferative autoimmune disorder in which keratinocytes form thick, scaly patches on the skin. Mouse IL-23-producing Langerhans cells are responsible for recruiting IL-17A-producing γδ T cells, which stimulate the growth of keratinocytes and lead to psoriatic sores (32). TNF-α produced by macrophages also stimulates γδ T cells in psoriatic environments, leading to an increase in IL-17A (33). Not only do local epidermal γδ T cells respond to these cytokines but circulating γδ T cells in the blood are also affected. In humans, Vγ9Vδ2 T cells are significantly fewer in number in psoriatic patients, as compared to healthy individuals (*n* = 32, *p* < 0.01) (34). Upon assessing the phenotype of Vγ9Vδ2 T cells, it was found that the number of cells carrying the pro-inflammatory cutaneous lymphocyte antigen (CLA^+^) skin-homing marker was reduced in patients with psoriasis compared to non-psoriatic controls, whereas the number of CLA^−^ Vγ9Vδ2 T cells was similar for each (34). The reduction in circulating Vγ9Vδ2 T cells with skin-homing receptors suggests that these cells have already infiltrated the skin. The harmful activity of γδ T cells in patients with psoriasis demonstrates how pro-inflammatory γδ T cells can potentially exacerbate chronic conditions and illustrates how future γδ-related treatments may face complex side effects.

### Inflammatory Bowel Disease

There are mixed reports about the role γδ T cells play in IBD. Research exploring the effects of IBD on peripheral blood mononuclear cells (PBMCs) in humans has demonstrated that although IBD patients exhibit no significant change in their number of activated CD3+ T cells, IBD patients possess a fourfold increase in circulating activated γδ T cells (35). γδ T cell activation in ulcerative colitis (UC) patient PBMCs was 2.5× greater than in controls without UC, and γδ T cell activation in Crohn’s disease patient PBMCs was over 10× greater than in controls (35). Further research found that a local increase in γδ T cell number is associated with IBD, as tissue samples collected from IBD-involved and non-involved regions of the same patients showed that IBD-involved regions experienced a fivefold increase in γδ T cells per gram of tissue (36).

A recent study compared gut-homing γδ T cells from healthy individuals with those of patients affected by UC and Crohn’s disease. Their findings indicated that patients with active UC and Crohn’s disease had a significant increase in the expression of C-C chemokine receptor type 9 (CCR9) (37). CCR9 (also known as CDw199) is a chemokine receptor and gut-homing marker located on circulating γδ T cells, which regulates cell migration. The same study also found that individuals with UC and Crohn’s disease had markedly reduced CD45RO (a protein tyrosine phosphatase and memory marker) expression on circulating γδ cells compared to healthy individuals (*p* < 0.0001). These circulating gut-homing cells may account for the common recurrence of UC and Crohn’s disease in patients (37).

### Asthma

Murine models of asthma suggest that IL-17A production by γδ T cells is anti-inflammatory and decreases the likelihood that a patient will experience airway hyperresponsiveness (AHR) (38). Another study found that the positive effect IL-17 has on the regulation of asthma in mice depends on the level of IL-17 attained (39). Low levels of IL-17 in conjunction with IL-13 are found to exacerbate asthma, while high levels of IL-17 with IL-13, are found to resolve the inflammation and resolve AHR (39). Patients with asthma have similar numbers of γδ T cells in their sputum, peripheral blood cells, and bronchoalveolar lavage as healthy patients, suggesting that asthmatic inflammation is not caused by an excess of γδ T cells (40). Given the variety of γδ T cells found throughout the body, it may be that multiple populations of γδ T cells interact to balance pulmonary IL-17 levels. Achieving a balance between γδ T cell populations that exacerbate inflammation and γδ T cell populations that resolve inflammation may be the key to controlling inflammation in the lungs.

## Conclusion

The innate and adaptive immune systems are bridged by γδ T cells, which serve as the guardians of the epithelium against trauma, infection, and other forms of damage. Epithelial barriers are critically important to the protection and health of the organism. γδ T cells are responsible for maintaining the homeostasis of the epithelium *via* the production of secreted factors, which can act in a paracrine manner to sustain a large impact. In this manner, γδ T cells not only modulate inflammation but also are sensitive to changes in the cytokine milieu caused by chronic inflammatory diseases. While cytokines produced by γδ T cells are necessary for the health of the tissues and cells, in high concentration these factors can yield chronic inflammation. Continued research is needed to elucidate the complexities of γδ T cell activation in chronic inflammation as well as the roles they play in disease pathogeny.

## Author Contributions

NF, EL, and JJ organized, wrote, and edited the manuscript. Figures were drawn by NF and edited by JJ.

## Conflict of Interest Statement

The authors declare that the research was conducted in the absence of any commercial or financial relationships that could be construed as a potential conflict of interest.

## Abbreviations

γδ T cells, gamma delta T lymphocytes; AHR, airway hyperresponsiveness; CAR, coxsackieviruses and adenovirus receptor; CCR9, C-C chemokine receptor type 9; CD100, cluster of differentiation 100; CD3, cluster of differentiation 3; CD45RA, cluster of differentiation 45RA; CLA, cutaneous lymphocyte antigen; DETC, dendritic epidermal T cells; Egr3, Early growth response 3; HFD, high fat diet; IBD, inflammatory bowel disease; IEL, intraepithelial lymphocyte; iNOS, inducible nitric oxide synthase; JAML, junction adhesive molecule-like protein; KGF-1, keratinocyte growth factor 1; MHC, major histocompatibility complex; NKG2D, NK receptor group 2 member D; PBMC, peripheral blood mononuclear cell; TCR, T cell receptor; TL1A, TNF-like protein 1A; TRAIL-R1, Tumor necrosis factor-related apoptosis-inducing ligand R1; TRAIL-R2, Tumor necrosis factor-related apoptosis-inducing ligand R2; UC, ulcerative colitis.
